# Intermittent tramadol vs tramadol administration via patient-controlled pump after lumbar discectomy: a randomized controlled trial

**DOI:** 10.3325/cmj.2022.63.110

**Published:** 2022-04

**Authors:** Biljana Kurtović, Krešimir Rotim, Tomislav Sajko, Cecilija Rotim, Adriano Friganović, Milan Milošević

**Affiliations:** 1 University of Applied Health Sciences, Zagreb, Croatia; 2 Department of Neurosurgery, Sestre Milosrdnice University Hospital Center, Zagreb, Croatia; 3 Rotim Polyclinic, Zagreb, Croatia; 4 Department of Anesthesiology and Intensive Medicine, University Hospital Center Zagreb, Zagreb, Croatia; 5 Department of Environmental Health, Occupational and Sports Medicine, Andrija Stampar School of Public Health, University of Zagreb School of Medicine, Zagreb, Croatia

## Abstract

**Aim:**

To compare the effect of intermittent tramadol dosing vs tramadol administration via patient-controlled pump on pain after lumbar discectomy.

**Methods:**

This randomized prospective study enrolled 100 patients who underwent elective LIV-LV lumbar discectomy in the neurosurgery department at Sestre Milosrdnice University Hospital Center from May 2016 to July 2017. Patients were randomized to receive either tramadol (600 mg daily) via a patient-controlled analgesia (PCA) pump or intermittently. Pain was evaluated by the Croatian version of Short-Form McGill Pain Questionnaire.

**Results:**

Forty percent of patients were women. The median (interquartile range) age of the patients was 51 (40-61) years. The groups did not differ in pain at 7 pm on the day of discectomy. However, in the morning and evening on the first postoperative day and in the morning and evening of the second postoperative day, the PCA group had significantly lower pain (*P* = 0.023, *P* < 0.001, *P* < 0.001, *P* = 0.026, respectively).

**Conclusion:**

This is the first study that used the Short Form McGill Pain Questionnaire to compare the effect of tramadol administration via PCA pump and intermittent administration on pain after LIV-LV discectomy in a neurosurgery department. Tramadol showed a good analgesic efficacy in lumbar spine surgery; tramadol via PCA controlled pain more effectively than intermittently administered tramadol.

Australian New Zealand Clinical Trials Registry: ANZCTR12618001893291.

Lower back pain due to lumbar disc herniation is a common complaint. In more than two-thirds of patients, non-medical pain relief methods did not decrease pain or even increased it (1). Lumbar discectomy is one of the most common spinal surgical procedures (2-4). The treatment of pain after lumbar discectomy includes specific analgesia administered according to pain intensity and balanced use of medications. Therefore, a successful treatment requires an understanding of neuroanatomy and of the complex pathological and physiological pain mechanisms.

Adequate pain management in the postoperative period demands an interdisciplinary approach and is associated with better functional outcomes, early patient mobilization, early hospital discharge, and preventing chronic pain occurrence (5,6). Unimodal analgesia implies administering one analgesic from a specific group (nonsteroidal anti-inflammatory drugs, opioids, non-opioids), requiring relatively high doses of analgesics to achieve a better pain relief.

Tramadol is an antinociceptive drug with low affinity for opioid receptors that achieves analgesia by combining indirect postsynaptic activation of two adrenergic receptors and opioid activity. It is administered at doses of up to 600 mg daily (7-13). Tramadol shows good analgesic efficacy and potency, comparable to codeine and morphine (14,15). It is associated with a lower risk of sedation and opioid addiction due to its low potency and weak affinity for μ receptors and blocking of repeated noradrenalin storage (16,17). A meta-analysis of 18 placebo-controlled studies confirmed the safety and efficacy of tramadol in treating moderate to severe postoperative pain, depending on the dose (18). However, some authors suggest that the relative “safety” of tramadol or opioids in general cannot be determined (19). Postoperative pain can be differently affected by different methods of unimodal analgesic administration. In some studies, patient-controlled analgesia (PCA), compared with conventional opioid analgesia, resulted in better pain control and increased patient satisfaction in postoperative pain management (20,21). While some studies indicated equal efficacy of intermittent and PCA administration in pain assessment (22,23), others showed PCA superiority (24). Unimodal administration of tramadol by two different methods has not been investigated in postoperative pain management after lumbar discectomy. The aim of this study was to investigate intermittent tramadol dosing vs tramadol administration via patient-controlled pump after lumbar discectomy.

## PATIENTS AND METHODS

### Design

**Figure 1:**
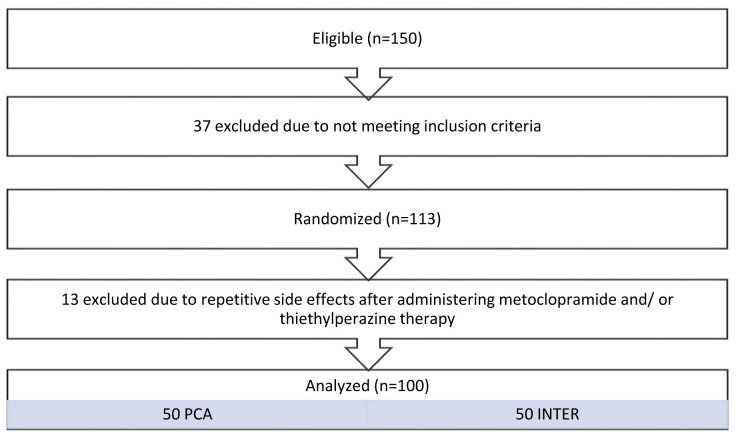
Patient recruitment flowchart.

In this prospective randomized study, 150 neurosurgical patients who underwent elective lumbar discectomy in the neurosurgery department of Sestre Milosrdnice University Hospital Center between May 2016 and July 2017 were considered eligible. The inclusion criteria were age older than 18; the first elective neurosurgical procedure on spine level LIV-LV; no analgesic allergies; no malignant or liver diseases; the mental capacity to give informed consent; the ability to use a PCA pump; and receiving tramadol as a postoperative pain relief. Thirty-seven patients were excluded as they did not meet the inclusion criteria. Overall, 113 patients were randomized to receive a 600 mg intravenous absolute dose of tramadol either applied by PCA pump or by intermittent IV administration. Randomization was performed by MedCalc for Windows, version 15.1 (www.medcalc.be). Since the trial investigators had no way of knowing which participant would go into which group, they could not influence the randomization. Group assignments were sealed within numbered envelopes given to nurses who administered the analgesia. Thirteen patients were excluded due repetitive side effects after administering metoclopramide (3 × 10 mg/24 hours intravenously) and/or thiethylperazine (2 × 6.5 mg/24 hours intravenously) therapy for nausea and vomiting. We did not collect the data on the number of excluded patients per group. Recruitment was continued until a target of 100 participants was reached (50 in each group) (Figure 1). Tramadol analgesia was prescribed by the attending physician and administered by nurses who were educated on the implementation of the study and methods of analgesia, with special reference to PCA application, before the start of the study. Pain was assessed with the Short-Form McGill Pain Questionnaire (SF-MPQ). This study is a part of a 200-patient study registered on Australian New Zealand Clinical Trials Registry (ANZCTR12618001893291). Tramadol 600 mg daily was administered postoperatively, either intermittently or via a PCA pump. Both groups used the same amount of tramadol, 600 mg/24 hours (Table 1). During lumbar discectomy, patients received general anesthesia with propofol (1.5-2.5 mg/kg), sufentanil (0.3 µg/kg), a vecuronium bolus dose (0.1 mg/kg), and sevoflurane 50%/50% O_2_.

**Table 1:** The characteristics of tramadol administration via patient-controlled analgesia (PCA) pump

Characteristic	
Drug dose per mL	12 mg/mL
Dilution volume	50 mL
Dose unit	mg/h
Soft limit	600 mg/24 h
Device type	Perfusor
Dose period	24 h
Set dose limits	600 mg
Bolus amount	20 mg
Lock time	120 minutes
Initial bolus	20 mg
Basal flow	1.38 mL/h

The sample size was calculated assuming an estimated postoperative difference of at least 40% more patients who do not feel pain at the end of the study in the PCA group compared with the INTER group. For the Fisher exact test with 95% of power, an alpha significance of 5%, and an equal number of patients in each group, the study required at least 41 patients per group.

### Instrument

Pain was assessed with the Croatian version of SF-MPQ in the evening on the day of the surgery, and in the morning and evening on the first and second postoperative day. The nurses received a one-hour theoretical education on the study topic and the SF-MPQ design with each questionnaire descriptor explained. The lead researcher supervised the daily SF-MPQ use for the first month of data collection to resolve any possible difficulties.

The patients signed an informed consent form for study participation. They were explained that they could withdraw their informed consent at any time during the study with no effects on their hospital treatment. The study was approved by the Ethics Committee of Sestre Milosrdnice University Hospital Center (EP-4433/15-9).

### Statistical analysis

Quantitative data are presented as arithmetic means, standard deviations, and 95% confidence intervals (CI). The Fisher-Freeman-Halton exact test was used for the analysis of differences in categorical variables between the groups. Cronbach α internal consistency coefficient was higher than 0.87, indicating appropriate scale structure of the questionnaire. The test groups scores are presented as scores on each answer ranging from “I don’t feel pain” to “I feel intolerable pain” (Table 2) and as a composite score (the sum of all 15 questions about the type of pain, Table 3). The composite score was obtained by adding up the results of all 15 items graded on a scale from 0 = I do not feel pain to 3 = I feel strong pain. The maximum score was 45, indicating the strongest level of experienced pain, while the minimum score was 0, indicating no pain. *p* values lower than 0.05 were considered significant. The analysis was performed with StatsDirect software, version 3.0.187. (StatsDirect Ltd, Wirral, UK).

**Table 2:** Short-Form McGill Pain Questionnaire (SF-MPQ) results in groups receiving intermittent tramadol (INTER) and tramadol via patient-controlled analgesia (PCA) pump

SF-MPQ		Group				Fisher-Freeman-Halton exact test (*p* values)
		INTER		PCA		
		No.	%	No.	%	
Preoperatively	I don’t feel pain	2	4	1	2	0.318
	I feel weak pain	5	10	2	4	
	I feel uncomfortable pain	13	26	18	36	
	I feel disturbing pain	19	38	21	42	
	I feel intolerable pain	11	22	8	16	
7 pm on the day of lumbar discectomy	I don’t feel pain	10	20	5	10	0.161
	I feel weak pain	18	36	15	30	
	I feel uncomfortable pain	14	28	21	42	
	I feel disturbing pain	8	16	8	16	
	I feel intolerable pain	0	0	1	2	
7 am on the first postoperative day	I don’t feel pain	8	16	16	32	0.023
	I feel weak pain	23	46	26	52	
	I feel uncomfortable pain	15	30	8	16	
	I feel disturbing pain	4	8	0	0	
	I feel intolerable pain	0	0	0	0	
7 pm on the first postoperative day	I don’t feel pain	8	16	21	42	<0.001
	I feel weak pain	22	44	25	50	
	I feel uncomfortable pain	18	36	4	8	
	I feel disturbing pain	2	4	0	0	
	I feel intolerable pain	0	0	0	0	
7 am on the second postoperative day	I don’t feel pain	9	18	33	66	<0.001
	I feel weak pain	25	50	17	34	
	I feel uncomfortable pain	14	28	0	0	
	I feel disturbing pain	2	4	0	0	
	I feel intolerable pain	0	0	0	0	
7 pm on the second postoperative day	I don’t feel pain	5	10.9	36	72	0.026
	I feel weak pain	34	73.9	13	26	
	I feel uncomfortable pain	7	15.2	0	0	
	I feel disturbing pain	0	0	1	2	
	I feel intolerable pain	0	0	0	0	

**Table 3:** The Short-Form McGill Pain Questionnaire (SF-MPQ) composite score in groups receiving intermittent tramadol (INTER) and tramadol via patient-controlled analgesia (PCA) pump (N = 50 in each group)

SF-MPQ		Arithmetic mean	Standard deviation	Standard error	95% confidence interval		Min	Max
					lower	upper		
Preoperatively	INTER	20.34	8.847	1.251	17.83	22.85	0	41
	PCA	18.52	9.283	1.313	15.88	21.16	0	38
7 pm on the day of the lumbar discectomy	INTER	9.28	6.459	0.913	7.44	11.12	1	38
	PCA	10.42	8.645	1.223	7.96	12.88	0	36
7 am on the first postoperative day	INTER	7.10	6.058	0.857	5.38	8.82	1	32
	PCA	5.28	5.410	0.765	3.74	6.82	0	23
7 pm on the first postoperative day	INTER	6.10	4.941	0.699	4.70	7.50	0	23
	PCA	3.22	4.752	0.672	1.87	4.57	0	23
7 am on the second postoperative day	INTER	5.02	4.162	0.589	3.84	6.20	0	18
	PCA	1.28	2.879	0.407	0.46	2.10	0	12
7 pm on the second postoperative day	INTER	4.04	3.849	0.544	2.95	5.13	0	17
	PCA	0.76	2.066	0.292	0.17	1.35	0	12

## RESULTS

The INTER and PCA group consisted of 50 participants each. The average hospital stay for each group was three days. In the PCA group, tramadol was administered as shown in Table 1. Intermitent tramadol was administered every six hours per day as 150 mg dose.

Of the 100 patients 40% were women. Median (interquartile range) age was 51 (40-61) years. The groups did not differ in demographic characteristics. The INTER group was significantly more physically active preoperatively (*P* = 0.001) (Table 4).

**Table 4:** Socio-demographic characteristics of groups receiving intermittent tramadol (INTER) and tramadol via patient-controlled analgesia (PCA) pump

		INTER		PCA	
		No.	%	No.	%
Age groups (years)	≤35	5	10	4	8
	35-45	11	22	13	26
	45-55	14	28	10	20
	55-65	13	26	14	28
	>65	7	14	9	18
Sex	male	30	60	30	60
	female	20	40	20	40
Occupation	unemployed	18	36	8	16
	moderate work	18	36	30	60
	exhausting work	14	28	12	24
Education	primary school	9	18	2	4
	secondary school	32	64	28	56
	community college	4	8	10	20
	university	5	10	10	20
Physically active	no	1	2	18	36
	yes	49	98	32	64
Smoking	no	29	58	34	68
	yes	21	42	16	32

At the first postoperative measurement, uncomfortable pain was felt by 28% of patients from the INTER group and by 42% of patients from the PCA group. At the second measurement, uncomfortable pain was reported by 30% and 16% patients, respectively; at the third measurement by 36% and 8%, respectively; at the fourth measurement by 28% and 0% respectively; and at the fifth measurement by 15.2% and 0% patients, respectively (Table 2). The PCA group had significantly lower pain at the second, third, fourth, and fifth measurement than the INTER group (Table 2).

## DISCUSSION

In our study, on the day of the surgery, 28% of the INTER group and 42% of the PCA group felt uncomfortable pain. After the second postoperative measurement, the PCA group reported significantly lower pain values. Hadi et al (25) showed that tramadol delivered by patient-controlled pump was as efficient as morphine administered by patient-controlled pump.

In our study, at the first postoperative measurement intermittently administered tramadol showed a superior analgesic efficacy compared with tramadol administered by a patient-controlled pump. However, at later measurements PCA group experienced better analgesic effect. This finding can be explained by the fact that a constant concentration of tramadol in plasma was achieved and the analgesic effect was maintained. The usual intermittent starting dose of tramadol is 100 mg twice a day, which, if the pain intensity does not decrease, has to be increased to 150/200 mg twice a day to a total maximum daily dose of 400 mg, and sometimes up to 600 mg. If administered intramuscularly or intravenously, the recommended dose is 50-100 mg, 4-6 times a day (13). In this study, the maximum dose of 600 mg/24 hours of tramadol was used. The safety and efficacy of dose-dependent tramadol in the treatment of moderate to severe postoperative pain were confirmed in a meta-analysis of 3453 patients in 18 placebo-controlled studies (18).

This study is limited by a lack of data collection on side effects of tramadol administration. Furthermore, we did not collect the data on the number of patients per group who were excluded due to repetitive side effects after administering metoclopramide and/or thiethylperazine therapy. We observed no erroneous use of PCA by the patients or any complications associated with PCA use.

This is the first study that uses the SF-MPQ to compare the effects of tramadol administration via PCA and intermittent tramadol administration on pain after LIV-LV discectomy in a neurosurgery department. Tramadol showed a good analgesic efficacy in lumbar spine surgery; PCA tramadol more effectively controlled pain than intermittent tramadol administration.
